# Whole‐Genome Analyses Reveal the Distinct Taxonomic Status of the Hainan Population of Endangered *Rucervus eldii* and Its Conservation Implications

**DOI:** 10.1111/eva.70010

**Published:** 2024-09-15

**Authors:** Chenqing Zheng, Qing Chen, Michelle Hang Gi Wong, Nick Marx, Thananh Khotpathoom, Hesheng Wang, Feng Yang, Xiaodong Rao, Bosco Pui Lok Chan, Yang Liu

**Affiliations:** ^1^ State Key Laboratory of Biocontrol, School of Life Sciences Sun Yat‐Sen University Guangzhou China; ^2^ School of Ecology Shenzhen Campus of Sun Yat‐Sen University Shenzhen China; ^3^ Kadoorie Farm and Botanic Garden Tai Po, New Territories Hong Kong China; ^4^ Wildlife Alliance Phnom Penh Cambodia; ^5^ Faculty of Forestry National University of Laos Vientiane Capital City Lao PDR; ^6^ Hainan Datian National Nature Reserve Dongfang City Hainan Province China; ^7^ School of Tropical Agriculture and Forestry Hainan University Danzhou China; ^8^ Haikou Key Laboratory of Intelligent Forestry Haikou China; ^9^ WWF‐Hong Kong Kwai Chung New Territories Hong Kong

**Keywords:** conservation management unit, genetic diversity, population genomics, translocation, ungulate

## Abstract

Eld's deer *Rucervus eldii* (McClelland, 1842) is an ungulate that lives in tropical lowland forests in several countries of Indochina and Hainan Island of China. Its remaining population is small and scattered, and the species is listed as an Endangered species on the IUCN Red List. The debate over the taxonomic status of the Hainan population has persisted for over a century—as an island‐endemic subspecies *R. e. hainanus*, or an insular population of the subspecies *R. e. siamensis*, would have significant conservation implications. And, given the Hainan population had experienced both population bottleneck and multiple translocations in the past, conservation genomics would be a powerful tool to evaluate the genetic impacts of these events. In this study, we used conservation genomics assessment to study population differentiation and genetic diversity of *R. e. siamensis* in Cambodia and three Eld's deer subpopulations on Hainan Island. Based on the unique genetic profile and demographic analysis, this study corroborated previous studies using genetic markers that the Hainan Eld's deer warrants the taxonomic status of a distinct subspecies. The Hainan population exhibits a reduction in genetic diversity and an increase in the level of inbreeding when compared to the population of Cambodia. The signs of purifying selection were found against homozygous loss‐of‐function mutations to decrease the deleterious burden in the Hainan population. However, there was an accumulation of more deleterious missense mutations. Furthermore, significant differences in genetic diversity and level of inbreeding found among the three Hainan subpopulations indicated population isolation and suboptimal translocation strategies, which calls for urgent, coordinated, and science‐based genetic management to ensure the long‐term viability of the endemic subspecies *hainanus*. This study provides guidance for the conservation and management of Eld's deer.

## 1 Introduction

Species rarity, endemism, and diversity are the major criteria for setting conservation priorities (Reid 1998); in addition, different subspecies often differ in geographic range and evolutionary potentials (Haig et al. 2006); therefore, infraspecific taxa are important in conservation planning (Mace 2004). Eld's deer *Rucervus eldii* (McClelland, 1842) is an endangered ungulate inhabiting dry lowland forest of tropical Asia. According to IUCN Red List and many literatures, the species comprised of three subspecies—the nominate subspecies is found in Manipur, India, subspecies *thamin* is distributed in the lowlands of the Tenasserim Hills of Myanmar and possibly westernmost Thailand, and subspecies *siamensis* is known from four Indochinese countries—Cambodia, Laos, Vietnam, and Thailand, as well as Hainan Island of China (Angom and Hussain 2013). All remnant populations are now small and threatened, and the populations in Vietnam and Thailand are now considered extirpated.

Based on a small number of skin and antler samples from Hainan, Lydekker (1915) first suggested the Hainan Eld's deer conspecific to the Eld's deer of Cambodia and Thailand and classified it as the subspecies *siamensis*. Later, Oldfield Thomas of the British Museum (Natural History) described the Hainan Eld's deer as smaller‐built with smaller and less elaborate antlers than those from Thailand, and classified it as an endemic subspecies *hainanus* (Thomas 1918). Using molecular approaches, several studies have found genetic divergence between the Hainan and Thailand populations (Angom 2012; Angom and Hussain 2013; Balakrishnan et al. 2003; Ghazi et al. 2021; Pumpitakkul et al. 2023; Zhang et al. 2009), but these results were inconclusive because of the use of maternally inherited mitochondrial DNAs, microsatellites and the repeated use of a small number of DNA sequences from GenBank (Wong, Mo, and Chan 2021). As a result, some earlier researchers, including the assessors of the IUCN Red List of Threatened Species, still consider the Hainan Eld's deer as a disjunct population of the subspecies *siamensis* (Gray et al. 2015; Groves and Grubb 2011).

Apart from taxonomy, conservation genomics can be a powerful tool to unveil both historical drivers and extant negative consequences of population declines (Formenti et al. 2022; Theissinger et al. 2023). Many endangered species have undergone dramatic population decline in the last decades mainly due to climate change (Boyce et al. 2022; Zhao et al. 2023), poaching (Minin et al. 2022; Kuiper et al. 2023), and habitat loss (Exposito‐Alonso et al. 2022; Sandor, Elphick, and Tingley 2022). Declining population size combined with isolation would impact population viability through intrinsic genetic depletion; in addition, the proportion of surviving and reproducing individuals can fluctuate sharply over time and so the chance of breeding between relatives increases (Berenos et al. 2016; Huisman et al. 2016). More importantly, this process can be exacerbated by genetic drift and inbreeding, which lead to further loss of genetic variability and the accumulation of deleterious alleles (Hedrick and Garcia‐Dorado 2016). The loss of genetic variation means a loss of adaptive potential (Ellegren and Galtier 2016), and the accumulation of deleterious alleles would reduce biological fitness (i.e. inbreeding depression) (Ochoa and Gibbs 2021). Although strongly deleterious mutations are effectively purged by natural selection, moderate deleterious mutations can be retained to influence the survival of small populations (Xie et al. 2022). Globally, the erosion of genetic variation and inbreeding are becoming major concerns in endangered species conservation (Amos and Balmford 2001).

Sharing the same fate as many other large mammals in this region, all remaining populations of Eld's deer are under severe threats of poaching and habitat loss. In particular, the Siamese Eld's deer (*R. e. siamensis*) was thought to be extinct in the wild across Indochina until its rediscovery in 1998 (McShea et al. 2005). On the other hand, the Hainan Eld's deer experienced a population crash with <50 individuals remaining in 1976, then gradually recovered to several hundred following drastic protection measures including fencing of prime habitats, reintroduction to extirpated range and translocation to bolster population size (Wong, Mo, and Chan 2021). Several studies using microsatellite and mitochondrial DNA (mtDNA) reported a low level of genetic variation in the Hainan Eld's deer (Pang et al. 2003; Zhang et al. 2005, 2008; Zhang, Ji, and Zeng 2007), indicating that strategies of translocating deer and establishing new subpopulations in the past failed to maintain genetic diversity. Maximizing genetic diversity and minimizing inbreeding and genetic drift are key management goals for establishing new or reintroduced populations (Frankham 1999; Ralls et al. 2020). Several studies proposed that translocation from multiple source populations (Nistelberger et al. 2023; Ransler, Quinn, and Oyler‐McCance 2010) and increasing population connectivity (DiLeo et al. 2017; Jangjoo et al. 2016; Malaney et al. 2017) would be effective measures to increase genetic diversity and abate founder effect. Further research with whole genome sequencing (WGS) is warranted to guide conservation efforts to boost genetic diversity and evolutionary potential (Mace 2004). Nonetheless, a recent study utilizing genome‐wide SNPs obtained through Restriction site‐associated DNA sequencing (RADseq) has provided new insights into the genomic diversity and demography of *R. e. siamensis* and *R. e. thamin* (Pumpitakkul et al. 2023). This information is highly helpful for the conservation management of Eld's deer, but it is urgently needed for the Indochinese and Hainan populations.

In this study, we carried out conservation genomic analysis on Eld's deer samples from Hainan of China (three subpopulations, blood samples), Cambodia (blood samples) and Laos (skin samples). Our study objectives were as follows: (1) to confirm the taxonomic status of Hainan Eld's deer; and (2) to assess the genetic diversity, inbreeding levels and mutation load of the three populations to inform conservation planning.

## 2 Materials and Methods

### 2.1 Sample Collection

**FIGURE 1:**
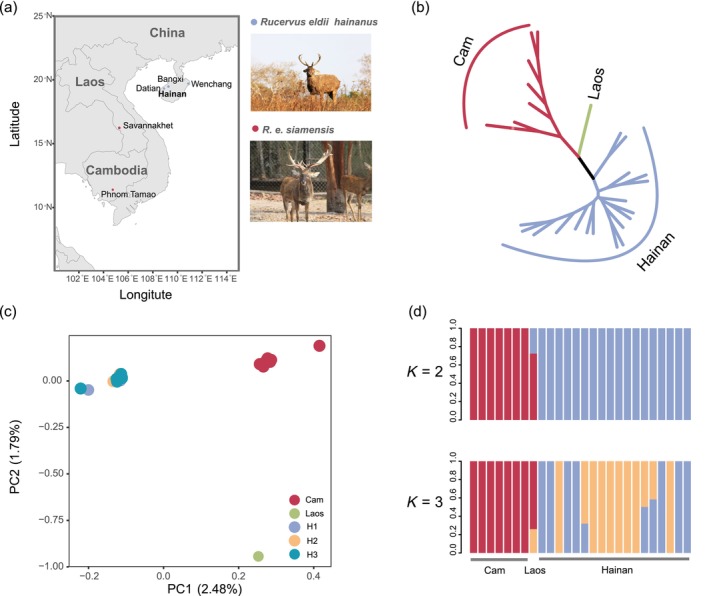
The sampling locations and genetic structure. (a) Map of sampling locations and the pictures of *Rucervus eldii hainanus* and *R. e. siamensis* provided by Funan Yun and Nick Marx of Wildlife Alliance, respectively. (b) Phylogenetic tree based on the maximum likelihood models. (c) Principal Component Analysis (PCA). Cam indicates the results for Cambodia (red), the result for Laos is represented by green, and H1, H2 and H3 represented the results for Bangxi (violet), Wenchang (yellow) and Datian (blue) respectively. (d) ADMIXTURE (*K* = 2 and 3). Colors denote individuals from the different geographical regions.

For Cambodia, 10 blood samples were collected from five males and five females in Phnom Tamao Zoological Park and Wildlife Rescue Centre in 2017 (Figure 1a). These individuals were descendants of two founders confiscated from the illegal wildlife trade in 2001; they represent the largest captive population of the country, and some individuals had already been released and bred naturally in the wild. The samples were stored in 100% ethanol and stored at low temperatures during transportation to the laboratory.

For China, 20 blood samples were obtained from Eld's deer in three isolated areas—eight samples were collected from Hainan Datian National Nature Reserve, six from Wenchang Eld's deer Management Station, and six from Bangxi Provincial Nature Reserve. Blood samples were obtained from rescued or habituated individuals in these semi‐captive facilities in 2016–2017. The samples were stored in 95% ethanol and stored at low temperatures during transportation.

For Laos, skin samples were obtained from three males and one female from the wild. One sample was collected by a local villager in 2002, and three samples were collected during 2017–2018 by researchers and reserve staff from fresh carcasses. The samples were in the form of dry skin of 100–200 cm^2^. As the Eld's deer population in Laos is estimated to be <100 individuals (Wong et al.  2018) and collecting blood samples from wild deer is impossible, these skin samples were the researchers' best option available.

### 2.2 DNA Extraction

DNA extraction from blood and dried skin samples was performed using DNeasy Blood and Tissue Kits (Qiagen, Germany), following manufacturer's guidelines. Dried skin samples were cleaned with 100% ethanol, air dried, and then incubated in Lysis Buffer overnight at 55°C. DNA concentrations were determined using NanoDrop 1000 Spectrophotometer (ThermoFisher Scientific, USA) and Qubit 2.0 Fluorometer (ThermoFisher Scientific, USA). DNA purity was measured by Nanodrop spectrophotometer and a ratio of A260/A280 between 1.8 and 2.0 indicates high‐quality DNA with minimal protein contamination. For building DNA libraries, a minimum concentration of 50 ng/μL measured by Qubit and a total yield of at least 1 μg for DNA amount were necessary.

### 2.3 Sequencing and Data Processing

The DNA libraries with 350 bp insert size were constructed and sequenced on Hiseq X ten in the Beijing Genomics Institute (BGI) company and Novogene company. Raw reads were filtered by removing adaptor sequences and low‐quality bases using default pipeline. We used the Burrows‐Wheeler Aligner (BWA) v0.7.6 (Li and Durbin 2009) with default parameters to align all clean data from each individual to the genome of the red deer *Cervus elaphus* (GCA_910594005.1). The aligned sequences were sorted using samtools v1.3.1 (Li et al. 2009), and duplicate marking was performed using picard tool in GATK v4.0.2.0 (McKenna et al. 2010). Bamdst (https://github.com/shiquan/bamdst) was used for obtaining the sequencing depth and coverage. Samples with <80% alignment have been removed, maintaining two samples in the Laos population, eight samples in the Cambodia population, 20 samples in the Hainan population for downstream analysis. GATK and vcftools v0.1.16 (Danecek et al. 2011) were used to identify the single nucleotide polymorphisms (SNPs). Low‐quality SNPs were removed based on these filtering criteria: (1) SQR >3 || FS >60 || MQ <40 || QD <2 || MQRankSum <−12.500 || ReadPosRankSum >8.000 || ReadPosRankSum <−8.000; (2) sequencing depth lower than 3, base‐missing rate higher than 10%, minimum quality values lower than 20, the Hardy–Weinberg expectation <0.05, minor allele frequency lower than 0.05. We removed the sites on the sex chromosomes based on the assembled complete reference genome and used autosomal SNPs for downstream analyses.

### 2.4 Population Differentiation

To analyze the genetic differentiation of the three populations, we used the autosomal SNPs to remove closely related individuals and strong linkage disequilibrium (LD) sites when conducting population structure analysis on the Hainan population. PLINK v1.9 (Purcell et al. 2007) was used to filter SNPs with a criterion of a LD coefficient (*r* ^2^ > 0.2) and KING v2.1.3 (Manichaikul et al. 2010) was applied to calculate the kinship coefficient. There were two samples with close relationships (kinship coefficient >0.177; duplicate samples/monozygotic twins, or first‐degree relatives) to other samples (Table S1, Figure S1) and were therefore removed. A total of 26 samples were retained in the subsequent analysis. We used three approaches to estimate the genetic structure of the remaining samples from Hainan, Cambodia and Laos. First, a phylogenetic tree was constructed using Phylip v3.2 (Felsenstein 2014) with 100 replicates based on the autosomal SNPs. Second, we used principal component analysis (PCA) to illustrate the genetic cluster by PLINK (Purcell et al. 2007) based on the same set. Third, the same set was carried out structure analyses with logistic prior and *K* range from 1 to 10 with 200 replicates to infer the number of ancestors of the Eld's deer using admixture v1.3.0 (Alexander and Lange 2011). We carried out a cross‐validation (CV) error plot to determine the optimal model of the value *K*.

### 2.5 Historical Population Dynamics

Because of low‐coverage sequencing data generated, the methods that need high‐quality genomic sequence data are not applicable for this study to reconstruct demographic history (Nadachowska‐Brzyska et al. 2016). We reconstructed the population trajectories based on the SNP frequency spectra (SFS) using Stairway plot2 (Liu and Fu 2020). The folded SFS was estimated using easySFS based on 28 individuals from the Hainan and Cambodia populations. The Stairway plot2 was implemented from 200 bootstrap SFS, the mutation rate was set to 2.11 × 10^−9^ per site per generation (Chen et al. 2019), and the generation time was set to 1.5 years (Wong, Mo, and Chan 2021). GONE (Santiago et al. 2020) was used to infer the very recent historical *N*_e_ based on the LD of all unrelated individuals. We used the average recombination rate of red deer (1.04 cM/Mb) as a reference for Eld's deer (Johnston et al. 2017). To detect whether there was gene flow occurred between the Hainan and Cambodia populations, we used fastsimcoal v2.7 (Excoffier et al. 2013) to infer the demography of the two populations based on folded SFS, applying three models during simulation: (a) no gene flow between the two populations, (b) gene flow occurred early after separation, and (c) gene flow occurred long after separation (Figure S2). For each model, 100,000 simulations and 40 optimization cycles were performed to estimate parameters and generate the maximum likelihood of each model. To identify the best‐fit model, the estimated maximum likelihood was used to calculate the Akaike information criterion (AIC) values.

### 2.6 Genetic Diversity

We estimated the genetic diversity of each population and subpopulation of Eld's deer by calculating the level of heterozygosity (*H*) and nucleotide diversity (*π*). The autosomal SNPs were filtered with depth of <4 and more 100 using the script (https://github.com/ZhengCQ/VcfFilter). The heterozygous sites of all individuals were estimated using vcftools v0.1.16 (Danecek et al. 2011) based on filtering SNPs. The values of *H* were calculated by heterozygous sites/effective genome length. The effective genome length was defined as the genome length covered by alignment. The values of *π* were estimated using vcftools v0.1.16 (Danecek et al. 2011) with 50‐kb windows and 20‐kb steps based on autosomal SNPs. The genetic diversity and subsequent genetic analyses eliminated the Laos population due to its low sample size. We have also collected published data of heterozygosity of 32 threatened mammalian species for comparing genetic diversity, including Chinese endemic species the giant panda (*Ailuropoda melanoleuca*), Yangtze finless porpoise (*Neophocaena asiaeorientalis*), and Baiji (*Lipotes vexillifer*) (Morin et al. 2021).

### 2.7 Assessment of Inbreeding Level

To assess the level of inbreeding, we calculated the number and length (>100 kb) of Run of Homozygosity (ROH) using PLINK v1.9 (Purcell et al. 2007) based on autosomal SNPs for each individual. The parameters were set as the following: ‐‐homozyg‐window‐snp 50 ‐‐homozyg‐snp 50 ‐‐homozyg‐window‐missing 3 ‐‐homozyg‐kb 100 ‐‐homozyg‐density 1000. We further computed the genomic inbreeding coefficient (*F*_ROH_) for each individual. *F*_ROH_ measured the fraction of the total length of ROH in genome effective length. The LD pattern was estimated using PopLDdecay v3.40 (Zhang et al. 2019).

### 2.8 Mutation Load Analysis

To explore the potential influence of high inbreeding in the Hainan Eld's deer, we utilized *R*_A/B_ to calculate the relative frequency of deleterious missense and loss‐of‐function (LoF) mutations as indicator of mutation load. First, the autosomal SNPs without filtering minor allele frequencies were annotated using SnpEff v4.380 (Cingolani et al. 2012). The mutations were classified into three types: missense mutation, synonymous mutations, and LoF mutations. Then, the impact of missense variants was defined according to Grantham's score (Grantham 1974; Li, Wu, and Luo 1984), a score for measuring the physical/chemical properties of amino acid changes. Deleterious and benign missense variants were defined as variants with Grantham's score >150 and <150, respectively. The deleterious variants' splice donor, splice acceptor start lost, stop lost, stop gained, and stop retained were determined as LoF variations. Finally, we used *R*_A/B_ method to estimate the relative frequency of derived deleterious alleles as in a study on the mountain gorillas (Xue et al. 2015). We calculated the derived homozygous and heterozygous sites in the Hainan population relative to the Cambodia population. The coding script brrAB we developed to calculate *R*_A/B_ (https://github.com/ZhengCQ/brrAB).

## 3 Results

### 3.1 Genetic Structure

We generated 30 resequencing genomes (Bangxi: *n* = 6, Wenchang: *n* = 6, Datian: *n* = 8, Cambodia: *n* = 8, Laos: *n* = 2), the average sequencing depth and coverage were 9.24 (2.13–19.83) × and 99.79 (99.70–99.92) %, respectively (Table S2). One previously published sequencing data (CAM003) (Leroux et al. 2023) of the Cambodia population was removed due to low sequencing depth (mean depth <3×). A total of 131,163 autosomal SNPs were obtained for downstream analysis through the alignment of resequencing data to the reference genome.

The genetic structure analysis was performed based on 27,529 SNPs from 26 DNA samples—18 from China, 7 from Cambodia, and 1 from Laos. The phylogenic tree separated China (refer to Bangxi, Wenchang and Datian of Hainan), Cambodia and Laos population as three distinct group (Figure 1b). The principal component analysis (PCA) also supported this division (Figure 1c). While *K* = 1 was shown to be the optimal value for admixture, it was observed that when *K* = 2, the China and Cambodia populations exhibited clear distinctions (Figure 1d, Figure S3). We observed moderate genetic difference between the China and Cambodia populations, mean *F*_st_ was 0.14 ± 0.13 (Table S3). In addition, shallow genetic differentiation was found among the three groups from Hainan, China (Bangxi, Wenchang, Datian). The mean *F*_st_ was 0.050 ± 0.073 between Bangxi and Wenchang, 0.063 ± 0.077 between Bangxi and Datian, and 0.054 ± 0.067 between Wenchang and Datian.

### 3.2 Demographic History

**FIGURE 2:**
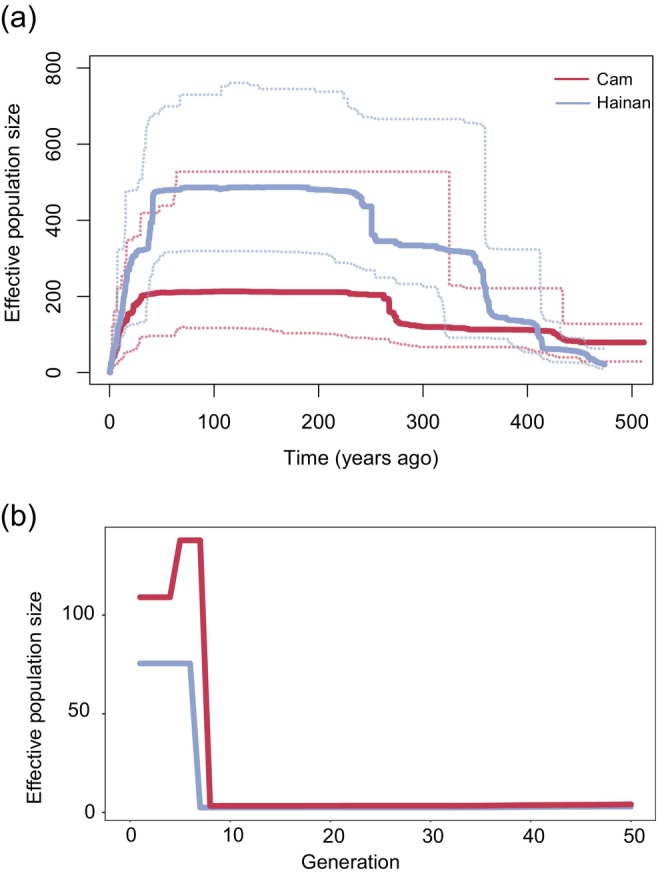
Demographic history of the study populations of *Rucervus eldii hainanus* and *R. e. siamensis*. (a) Inference of recent demographic history using Stairway plot2. (b) The inference of effective population size using GONE. Cam represents the Cambodia population and Hainan represents the three Hainan subpopulations. The dashed line represents the confidence interval of 90%–95%.

To explore the population trajectories, we reconstructed the demographies of the China and Cambodia populations using the Stairway plot2 (Figure 2a) and GONE (Figure 2b). The results of Stairway plot2 showed that both effective population sizes increased from 800 years ago and the *N*_e_ declined to <200 during the last 200 years. The results of GONE showed that the *N*_e_ of the two populations increased in the last 10 generations, the *N*_e_ of the Cambodian population maintained at approximately 110, and the *N*_e_ of the China population maintained at approximately 70. The best model (Table S4) using fastsimcoal v2.7 showed that the two populations diverged at about 27,288 years ago (CI 95%: 17,078–64,422 years), and there was gene flow at the early stage of divergence. The migration rate from the Cambodia population to the China population was 0.0129 (CI 95%: 0.0094–0.0619), and the reverse migration rate was 0.0200 (CI 95%: 0.0140–0.0912).

### 3.3 Genetic Diversity and Inbreeding

**FIGURE 3:**
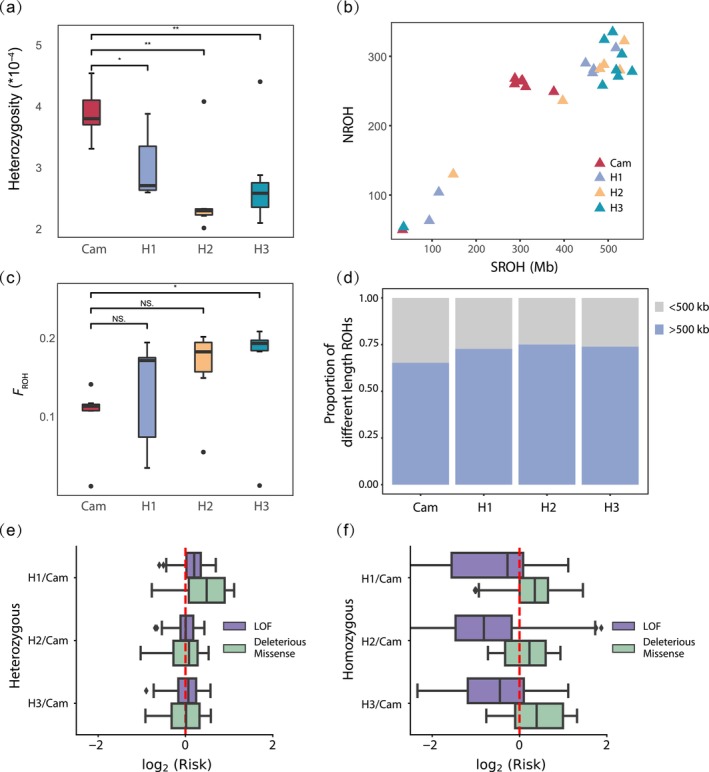
Genetic diversity, ROHs and mutation load of the study populations of *Rucervus eldii hainanus* and *R. e. siamensis*. (a) The heterozygosity of the Cambodia population and the Hainan population. (b) The length (SROH) and number (NROH) of ROH. (c) The inbreeding coefficient (*F*_ROH_), the ratio of ROH length and autosomal genome length. Cam indicates the results for Cambodia (red), and H1, H2 and H3 represented the results for Bangxi (violet), Wenchang (yellow) and Datian (blue) respectively. (d) The ratio of ROH > 500 and < 500 kb. (e, f) The relative genetic load risk scores for H1, H2, and H3 correspond to the results of Bangxi, Wenchang, and Datian, respectively, based on either heterozygous (e) or homozygous (f) genetic variants, in contrast to the data from Cambodia. *significant with *p* < 0.05; **significant with *p* < 0.01; NS, non‐significant.

The mean heterozygosity of the Cambodia population (3.92 × 10^−4^ ± 4.02 × 10^−5^) was significantly higher than that of the China population (2.78 × 10^−4^ ± 6.76 × 10^−5^) (Figure 3a). Overall, the mean nucleotide diversity (*π*) of the Cambodia population (6.84 × 10^−5^ ± 3.29 × 10^−4^) was higher than that of the China population (6.77 × 10^−5^ ± 2.88 × 10^−4^), but lower than each of the subpopulations (Bangxi: 8.98 × 10^−5^ ± 3.22 × 10^−4^, Wenchang: 9.42 × 10^−5^ ± 3.56 × 10^−4^, Datian: 8.15 × 10^−5^ ± 3.11 × 10^−4^), indicating greater genetic difference among the Hainan subpopulations than that between the China and Cambodia populations. Compared to published data for 32 threatened mammalian species, Eld's deer is among the nine species with the lowest genetic diversity (Figure S4). In particular, the mean heterozygosity of the China population and the Cambodian population of Eld's deer was even lower than that of the giant panda (1.35 × 10^−3^) and the Yangtze finless porpoise (1.05 × 10^−3^), while it is slightly higher than that of the Baiji (1.20 × 10^−4^) (Figure S4, Table S5).

We found that the number and length of ROH of the China population were higher than those of Cambodia (Figure 3b). The China population had an average number of ROH of 248 ± 86 and average length of ROH of 416.96 ± 168.33 Mb, whereas the Cambodia population had an average number of ROH of 230 ± 79 and average length of ROH of 272.30 ± 109.72 Mb. The genomic inbreeding coefficient, *F*_ROH_, was significantly higher for the China population than in the Cambodia population (Figure 3c). Most of ROHs were more than 500 kb among the populations (China: 73.93%, Cambodia: 65.26%; Figure 3d). The China population had longer ROHs compared to that of Cambodia, which indicated that this population underwent inbreeding events more recently. Moreover, the China population has a slow LD decay to the Cambodia population as shown in the autosome genome (Figure S5). These results indicate that a higher level of inbreeding occurred in the China population relative to the Cambodia population.

### 3.4 Mutation Load

We found a significantly greater divergence of relative frequency in the heterozygous LoF mutation forms among the three Hainan subpopulations (Figure 3e). Compared with the Cambodian population, the three Hainan subpopulations exhibited an excess of heterozygous deleterious missense and LoF mutations. Additionally, the three Hainan subpopulations showed similar homozygous LoF mutation counts, and a higher frequency of homozygous deleterious missense mutations relative to the Cambodian population (Figure 3f). However, the LoF mutations of the three Hainan subpopulations were relatively less in the homozygous forms than the Cambodia population. This result suggests that although the China population has more accumulation of deleterious mutations than the Cambodia population due to a constantly small population, strongly deleterious mutations (LoF mutations) could have been purged during bottleneck events.

## 4 Discussion

Empirical studies suggested that stochastic environmental factors, demographic events, and genetic processes would vastly impact the genetic viability of populations (Diez‐Del‐Molino et al. 2018; Melbourne and Hastings 2008). Eld's deer has experienced significant range contractions and local extinctions, which would undoubtedly cause changes in its genetic composition. Hence, genetic data are particularly pertinent to the conservation and management of Eld's deer. This study sheds light on the importance of applying genomic approaches to understand taxonomy and demographic history, providing a basis for effective conservation and management of an endangered species.

Our demographic analysis estimated that the China and Cambodia populations split at approximately 27,288 years ago and gene flow occurred in the early stage of divergence. This result supported the claim that the Hainan Eld's deer spread from Indochina to Hainan Island through a land bridge during the Pleistocene glacial age (Zhang et al. 2009). In South‐East Asia, there were significant glacio‐eustatic sea level fluctuations during the Middle and Late Pleistocene, when sea level dropped by 50–150 m (Tougard 2001; Voris 2000). This led to a land bridge connection between mainland South‐East Asia and Hainan Island and provided a possible colonization pathway for organisms from Indochina. It is noteworthy that the estimated divergence time between *R. e. siamensis* and *R. e. hainanus* in this work is approximately 27,000 years ago (Kya), which is significantly younger than the estimates from other studies, which were around 200 Kya (Ghazi et al. 2021; Pumpitakkul et al. 2023). This discrepancy is not unexpected given that the assessment of divergence time using phylogenetic methods and population scaled analysis depends on models of nucleotide substitution and the coalescent model, respectively (Arbogast et al. 2002). Moreover, it is likely that migration between populations typically leads to an underestimation of divergence times (Leaché et al. 2014). Based on phylogenetic analysis, PCA and admixture analysis, the China and Cambodia populations of Eld's deer became isolated and diverged. This finding is corroborated from the previous phylogenetic results that these two subspecies formed monophyletic clades against the sister clade comprising *R. e. eldii* and *R. e. thami* (Pumpitakkul et al. 2023). Population divergence using *F*_st_ indicated a significant genetic differentiation now exists between the China and Cambodia populations, as found in some previous studies based on mtDNA sequences (Balakrishnan et al. 2003; Zhang et al. 2009). The divergence implied that the Hainan Eld's deer had experienced founder events and genetic drift at least since the end of the Pleistocene and early Holocene (18,000–8500 years ago) when sea level rose and Hainan became an isolated island (Balakrishnan et al. 2003). Other evidences of such geographic connection include some vertebrate groups such as the Hainan and Taiwan partridges (*Arborophila ardens and A. crudigularis*) were found to have originated from Indochina (Chen et al. 2015); the Hainan gymnure *Neohylomys hainanensis* was also discovered in Vietnam (Abramov et al. 2018); and the largescale silver carp *Hypophthalmichthys harmandi* only occur on Hainan Island and the Red River of Vietnam (Froese and Pauly 2020).

The isolation of the Hainan Eld's deer caused significant morphological divergence from its counterparts in southeast Asia. Apart from the description by Oldfield Thomas (1918), other data indicate that the Hainan Eld's deer is indeed markedly smaller than the mainland Siamese Eld's deer in terms of body mass and size (Wong, Mo, and Chan 2021), and in terms of its antlers, the Hainan Eld's deer is considered to have less elaborate antlers than the mainland form, where small snags above the pedicle and palmation are both rare (Wong, Mo, and Chan 2021). Nevertheless, the genome‐wide divergence was found to be lower between *R. e. siamensis* and Hainan Eld's deer than at the interspecific level based on the analyses of mitogenomes (Pumpitakkul et al. 2023). Collectively, these results suggest that the Hainan populations are on independent evolutionary trajectories and are in the early stage of speciation.

It has been documented that from the Ming and Qing dynasties, increased hunting activities have caused Hainan Eld's deer to undergo quick and dramatic range contraction, population decrease, and extirpation (Zeng et al. 2005). Since 1976, in situ conservation has been implemented to protect the remaining 26 individuals in Datian National Nature Reserve (Zhang et al. 2008). In 1990–1992, 19 deer were translocated from Datian to Bangxi, and in 2000, 22 deer were translocated from Datian to establish a captive population in Wenchang County. It was suggested that the first relocation to Bangxi comprised of four males and one female (i.e., one breeding pair) could have significantly limited the effective size of the founder population, leading to low genetic variability (Zhang et al. 2008). On the other hand, the translocation of 16 deer from another translocated population (i.e., the deer in the Hainan Tropical Wildlife Park originated from Datian) to Wenchang would have led to double founder effects, also leading to low genetic variability. We found weak genetic differences in the three sampling locations on Hainan Island. For instance, we found that individuals from Bangxi and Wenchang have generated slight genetic differentiation in the past 30 years due to geographical isolation and perhaps genetic drift. Small effective population size owing to the long‐term population decline and isolation has increased inbreeding and reduced genetic diversity of the Eld's deer subpopulations on Hainan. This result is consistent with a previous study using microsatellite DNA loci (Zhang et al. 2008).

The Eld's deer populations in Cambodia and China both showed significant degradation in genetic diversity. Meanwhile, the Hainan subpopulations showed significantly lower genetic diversity than that of the Cambodia population. The low genetic diversity of the Hainan subpopulations could be the consequence of splitting the herds into isolated subpopulations for multiple generations as discussed above, and the restricted gene exchange would have led to an increase in genetic drift and inbreeding depression than a coherent population that are able to breed freely. The inconsistent levels of genetic diversity among the Hainan subpopulations further suggested founder effects and genetic drift from the splitting of the population. Such phenomenon was also observed in the reintroduced Svalbard reindeer (*Rangifer tarandus platyrhynchus*) (Burnett et al. 2023), Eurasian lynx (*Lynx lynx*) (Mueller et al. 2022), and sika deer (*Cervus nippon*) of the Delmarva Peninsula, USA (Kalb et al. 2019). The change in genetic diversity from founding events can be affected by the rate and number of population breeding, genetic drift, and other stochastic events (Jamieson 2011). The differences of genetic diversity of the Bangxi and Wenchang subpopulations can be explained by the different ways of reintroduction—the Bangxi subpopulation was reintroduced from multiple batches of a small number of individuals, and the Wenchang subpopulation was originated from two source locations. Our results indicated that the Hainan and Cambodia populations have generated longer and more ROHs due to inbreeding. The inbreeding level observed in the China population was higher than that of the Cambodia population, and the long‐term survival of the China population is still at risk of inbreeding depression. Inbreeding depression is caused by the accumulation of deleterious mutations reducing the fitness of populations (Dussex et al. 2023). Even though the three Hainan subpopulations had significantly more deleterious mutations compared to the Cambodia population, the most damaging mutations (LoF mutations) are relatively less abundant, which could be the result of purging for highly deleterious mutations and so the impact of inbreeding depression is reduced. Compared to the Cambodia population, the three Hainan subpopulations have more common heterozygote genotypes in deleterious mutations (deleterious missense mutations and LoF mutations). Empirical evidence suggests that the impact of recessive mutations is low unless they are expressed (Agrawal and Whitlock 2011). In the case of Hainan Eld's deer, long‐term isolation of small populations could increase the level of inbreeding, promote the expression of the recessive mutations and thereby reduces population fitness and long‐term viability of the population.

It is worth noting that the sample size and sequencing depth are limited in the study. The decision to use low‐coverage sequencing for this study was based on its cost‐effectiveness. However, it has been demonstrated that this resequencing strategy is sufficient for making reliable inferences on genome‐wide diversity and historical population demography (Lou et al. 2021). Importantly, we carried out multiple processes to simultaneously identify genetic variations and accurately genotype individuals with high confidence. We also carefully chose demographic modeling methods that are capable of accurately inferring historical demography without the need for high‐coverage sequencing data. These efforts enable us to obtain robust and timely conservation implications of *Rucervus eldii*.

In conclusion, this is the first genomic study on the population divergence of Hainan Eld's deer and its conspecifics in Indochina. Based on the genome‐wide differentiation identified in this study and differences in morphological traits, we recommended *Rucervus eldii hainanus* is a valid and endemic subspecies in Hainan Island, China. Given the high level of inbreeding and low level of genetic diversity among the three Hainan subpopulations, we suggested a regular exchange of individuals between source (e.g., Datian) and translocated subpopulations, and only individuals from Datian should be used to establish new subpopulations to maximize genetic diversity and maintain the long‐term viability of the subspecies. Nevertheless, individual exchange among subpopulations needs to be prudently performed, because local adaptation and genetic load may contribute to outbreeding depression. In our study, we found a weak genetic differentiation but significant load difference of deleterious mutations among the Hainan subpopulations. Undoubtedly, further analysis on the mechanism of local adaptation among the Hainan subpopulations with a larger sample size and deep sequencing is recommended. Furthermore, to monitor for outbreeding depression, exchanged individuals and their offspring should be marked and kept in a restricted range, and their survival rate and genetic indicators should be closely monitored. However, it is pertinent to be cautious when interpreting any indications of inbreeding and outbreeding depression in the absence of extensive field evidence.

## Conflicts of Interest

The authors declare no conflicts of interest.

## Supporting information


Appendix S1.

## Data Availability

Data for this study are available in the National Genomics Data Center under BioProject PRJCA016618 (accession numbers of each sample: SAMC1224803‐30).
