# Supplementary material for: Whole‐Genome Analyses Reveal the Distinct Taxonomic Status of the Hainan Population of Endangered Rucervus eldii and Its Conservation Implications
Source: Evol Appl. 2024 Sep 15;17(9):e70010. doi: 10.1111/eva.70010 (PMC11403188; doi:10.1111/eva.70010)
Supplement: Supplementary file 1 — Appendix S1. [file EVA-17-e70010-s001.zip › eva70010-sup-0001-Supinfo.docx]

**Supplementary materials**

**Whole-genome analyses reveal the distinct taxonomic status of the Hainan population of Endangered *Rucervus eldii* and its conservation implications**


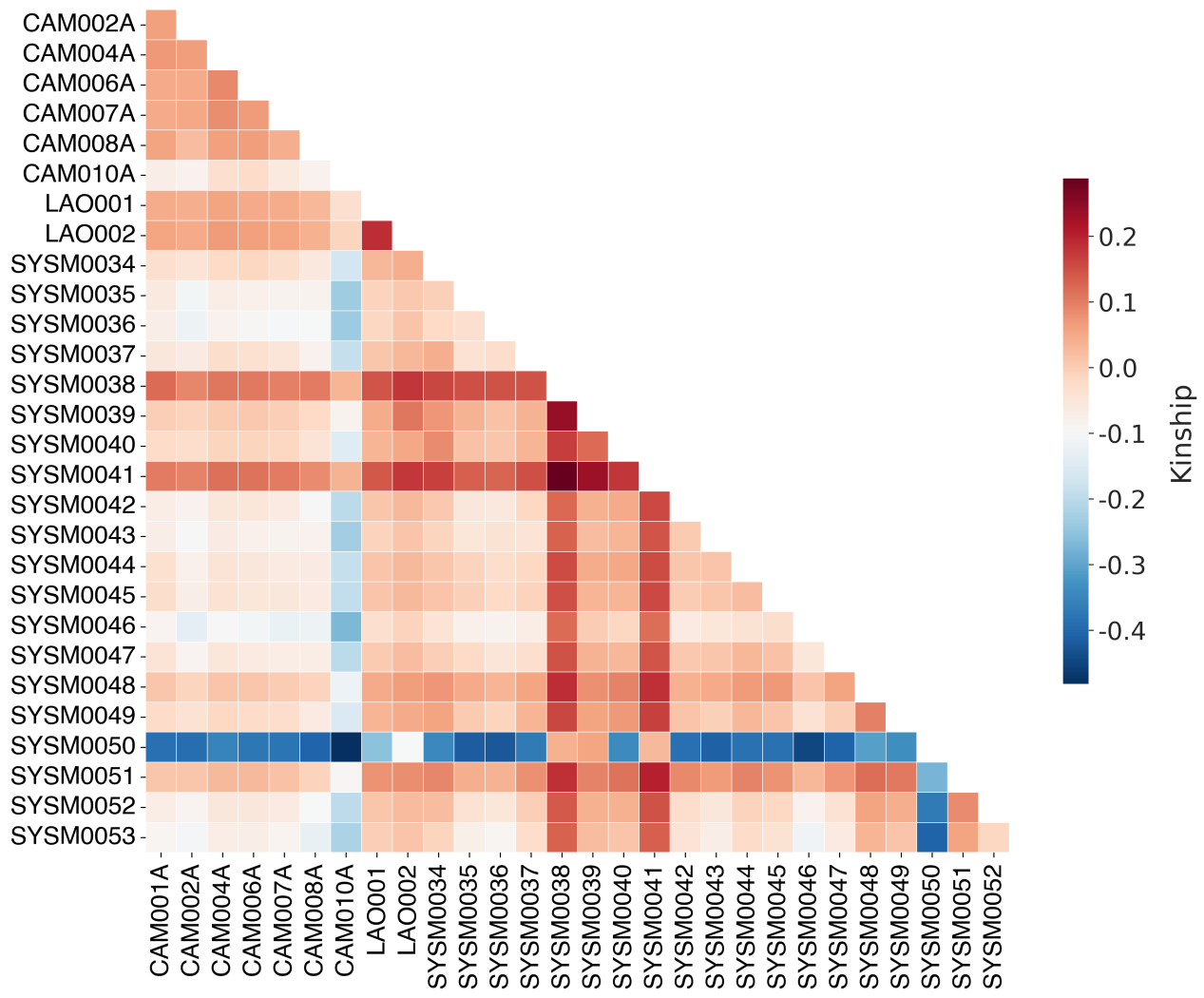


Figure S1 The heatmap of kinship coefficient among all of 28 samples from populations of *Rucervus eldii hainanus* and *Rucervus eldii siamensis* using KING v2.1.3.


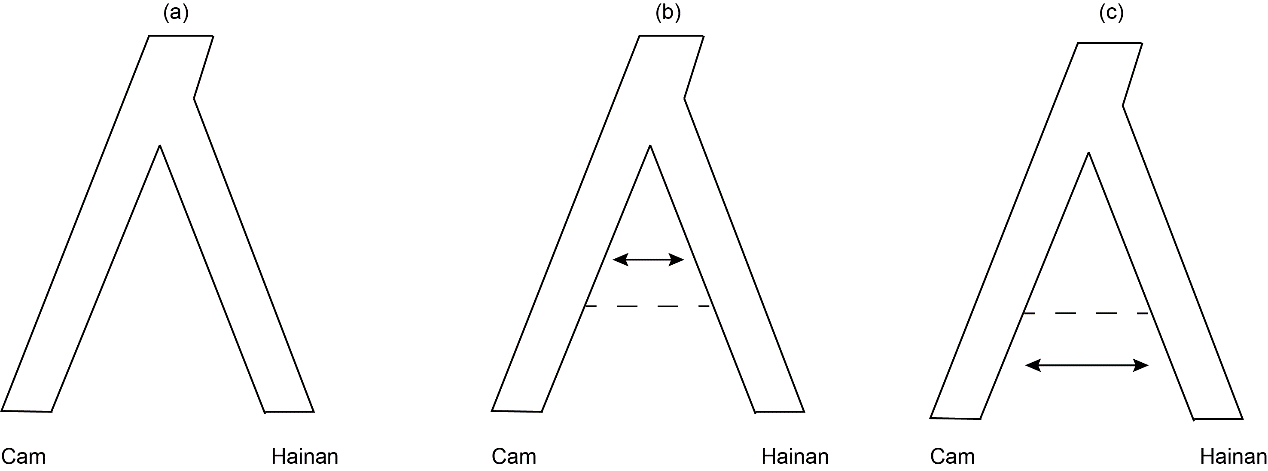


Figure S2 Population demographic history models of populations of *Rucervus eldii hainanus* and *Rucervus eldii siamensis*, simulated using fastsimcoal v2.7. (a) indicates that there was no gene flow between populations (b) indicates that there was gene flow at the early stages between populations (c) indicates that there was gene flow at the recent stages between populations


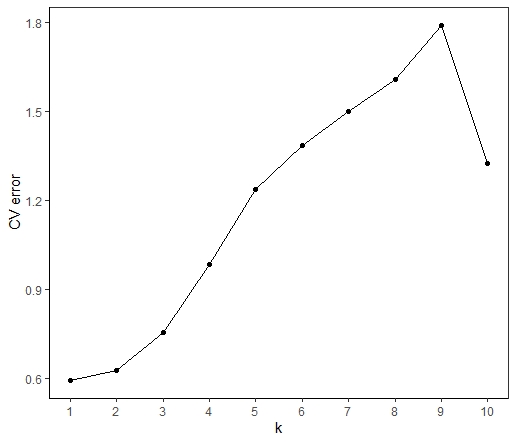


Figure S3 The cross-validation (CV) error values were calculated for K values ranging from 1 to 10. The optimal K, representing the number of ancestors, was identified as 1, corresponding to the lowest CV error value.


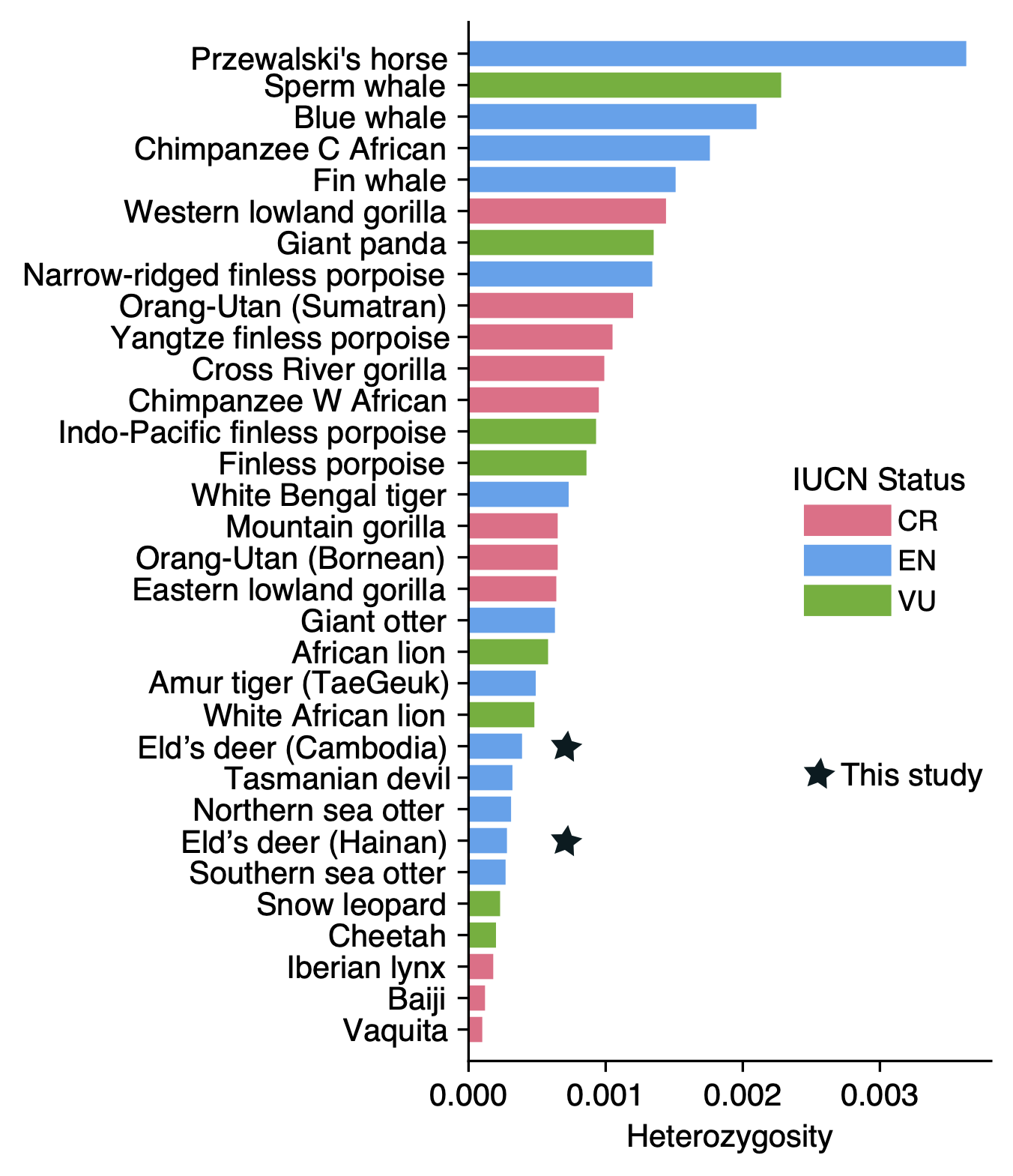


Figure S4 Comparison of mean genome-wide diversity in *Rucervus eldii hainanus* (Chinese population) and *Rucervus eldii siamensis* (Cambodia population) for other threatened mammalian species.


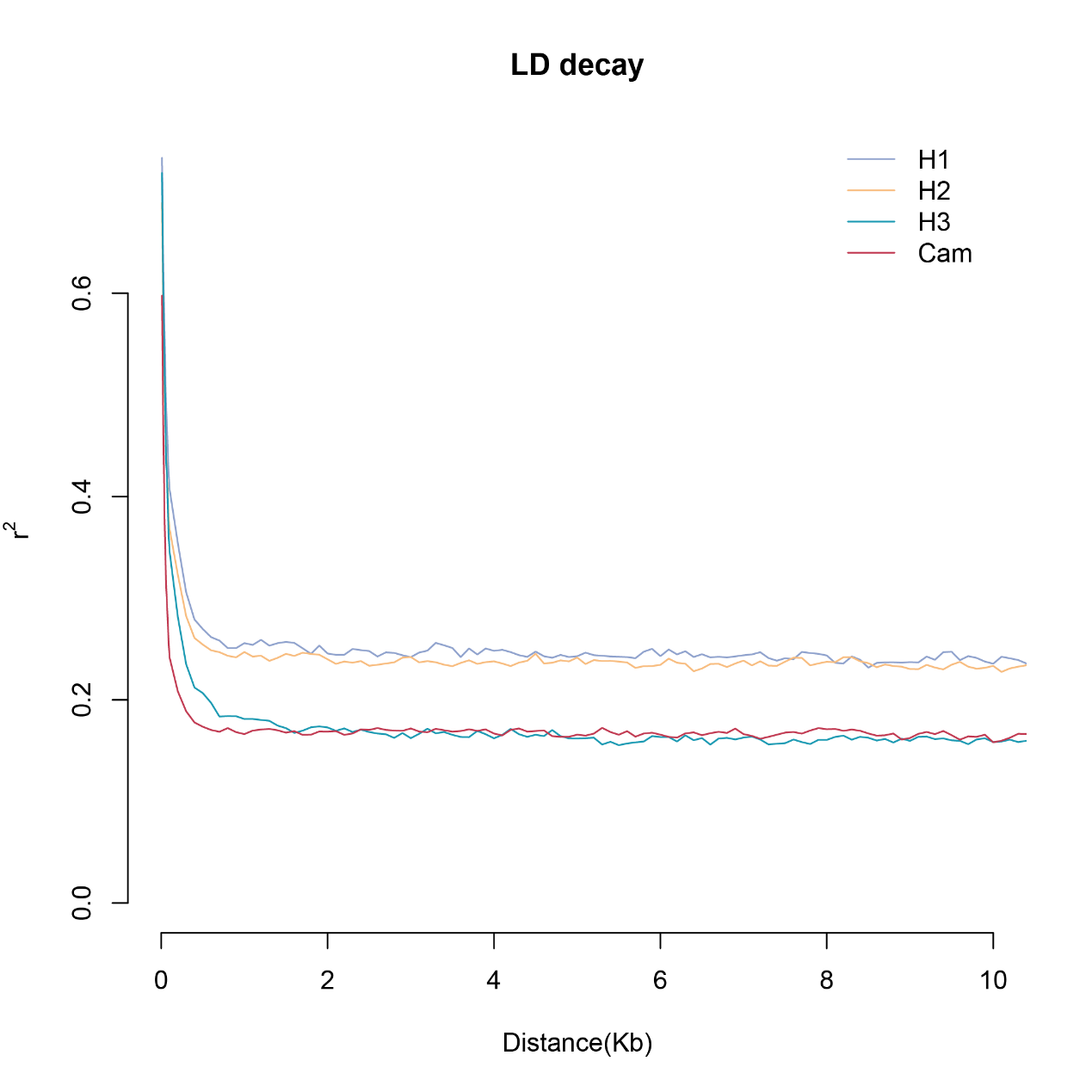


Figure S5 Linkage Disequilibrium (LD) decay of *Rucervus eldii hainanus* (Hainan) and *Rucervus eldii siamensis* (Cambodia) based on autosome chromosomes.

Supplementary Tables

Table S2 The summary of sequencing depth and coverage for each sample of *Rucervus eldii hainanus* and *R. e. siamensis*,

| **sample** | **depth** | **coverage** |
| --- | --- | --- |
| CAM001 | 7.44 | 99.83% |
| CAM002 | 8.00 | 99.82% |
| CAM003 | 2.13 | 99.70% |
| CAM004 | 8.94 | 99.83% |
| CAM006 | 8.59 | 99.82% |
| CAM007 | 8.66 | 99.79% |
| CAM008 | 7.50 | 99.83% |
| CAM0010 | 5.54 | 99.77% |
| SYSU0034 | 8.02 | 99.77% |
| SYSU0035 | 7.05 | 99.82% |
| SYSU0036 | 6.43 | 99.83% |
| SYSU0037 | 8.01 | 99.81% |
| SYSU0038 | 19.83 | 99.85% |
| SYSU0039 | 19.81 | 98.92% |
| SYSU0040 | 11.16 | 99.54% |
| SYSU0041 | 17.79 | 99.86% |
| SYSU0042 | 9.55 | 99.77% |
| SYSU0043 | 7.88 | 99.77% |
| SYSU0044 | 8.42 | 99.79% |
| SYSU0045 | 8.11 | 99.79% |
| SYSU0046 | 7.44 | 99.77% |
| SYSU0047 | 8.54 | 99.78% |
| SYSU0048 | 8.47 | 99.80% |
| SYSU0049 | 9.12 | 99.77% |
| SYSU0050 | 8.14 | 99.76% |
| SYSU0051 | 11.18 | 99.80% |
| SYSU0052 | 8.72 | 99.75% |
| SYSU0053 | 8.11 | 99.78% |
| Laos01 | 4.07 | 99.79% |
| Laos02 | 3.96 | 99.77% |

Table S3 The mean pairwise *F*_st_ based on all populations of *Rucervus eldii hainanus* and *R. e. siamensis*

| Group | F_st_ |
| --- | --- |
| Cambodia-Hainan (all) | 0.138 |
| Datian-Bangxi | 0.050 |
| Datian-Wenchang | 0.054 |
| Bangxi-Wenchang | 0.050 |

Table S4 The model results of Fastsimcoal v2.7

| Model | Parameters(d) | MaxEstLhood | MaxObsLhood | AIC |
| --- | --- | --- | --- | --- |
| (a) | 4 | -59034.359 | -22494.371 | 118076.718 |
| **(b)** | **7** | **-23358.246** | **-22494.371** | **46730.492** |
| (c) | 7 | -58718.625 | -22494.371 | 117451.25 |
